# A comparison of conventional and advanced 3D imaging techniques for percutaneous left atrial appendage closure

**DOI:** 10.3389/fcvm.2024.1328906

**Published:** 2024-03-26

**Authors:** Houtan Heidari, Dominika Kanschik, Oliver Maier, Georg Wolff, Maximilian Brockmeyer, Maryna Masyuk, Raphael Romano Bruno, Amin Polzin, Ralf Erkens, Gerald Antoch, Sebastian Daniel Reinartz, Nikos Werner, Malte Kelm, Tobias Zeus, Shazia Afzal, Christian Jung

**Affiliations:** ^1^ Division of Cardiology, Pulmonology and Vascular Medicine, Heinrich Heine University, Medical Faculty, Düsseldorf, Germany; ^2^ Department of Diagnostic and Interventional Radiology, University Düsseldorf, Medical Faculty, Düsseldorf, Germany; ^3^ Department of Cardiology, Heartcenter Trier, Krankenhaus der Barmherzigen Brüder, Trier, Germany; ^4^ CARID (Cardiovascular Research Institute Düsseldorf), Düsseldorf, Germany

**Keywords:** left atrial appendage closure, virtual reality, cardiac computed tomography, 3D printing, transesophageal echocardiography

## Abstract

**Background:**

Understanding complex cardiac anatomy is essential for percutaneous left atrial appendage (LAA) closure. Conventional multi-slice computed tomography (MSCT) and transesophageal echocardiography (TEE) are now supported by advanced 3D printing and virtual reality (VR) techniques for three-dimensional visualization of volumetric data sets. This study aimed to investigate their added value for LAA closure procedures.

**Methods:**

Ten patients scheduled for interventional LAA closure were evaluated with MSCT and TEE. Patient-specific 3D printings and VR models were fabricated based on MSCT data. Ten cardiologists then comparatively assessed LAA anatomy and its procedure relevant surrounding structures with all four imaging modalities and rated their procedural utility on a 5-point Likert scale questionnaire (from 1 = strongly agree to 5 = strongly disagree).

**Results:**

Device sizing was rated highest in MSCT (MSCT: 1.9 ± 0.8; TEE: 2.6 ± 0.9; 3D printing: 2.5 ± 1.0; VR: 2.5 ± 1.1; *p* < 0.01); TEE, VR, and 3D printing were superior in the visualization of the Fossa ovalis compared to MSCT (MSCT: 3.3 ± 1.4; TEE: 2.2 ± 1.3; 3D printing: 2.2 ± 1.4; VR: 1.9 ± 1.3; all *p* < 0.01). The major strength of VR and 3D printing techniques was a superior depth perception (VR: 1.6 ± 0.5; 3D printing: 1.8 ± 0.4; TEE: 2.9 ± 0.7; MSCT: 2.6 ± 0.8; *p* < 0.01). The visualization of extracardiac structures was rated less accurate in TEE than MSCT (TEE: 2.6 ± 0.9; MSCT: 1.9 ± 0.8, *p* < 0.01). However, 3D printing and VR insufficiently visualized extracardiac structures in the present study.

**Conclusion:**

A true 3D visualization in VR or 3D printing provides an additional value in the evaluation of the LAA for the planning of percutaneous closure. In particular, the superior perception of depth was seen as a strength of a 3D visualization. This may contribute to a better overall understanding of the anatomy. Clinical studies are needed to evaluate whether a more comprehensive understanding through advanced multimodal imaging of patient-specific anatomy using VR may translate into improved procedural outcomes.

## Graphical Abstract

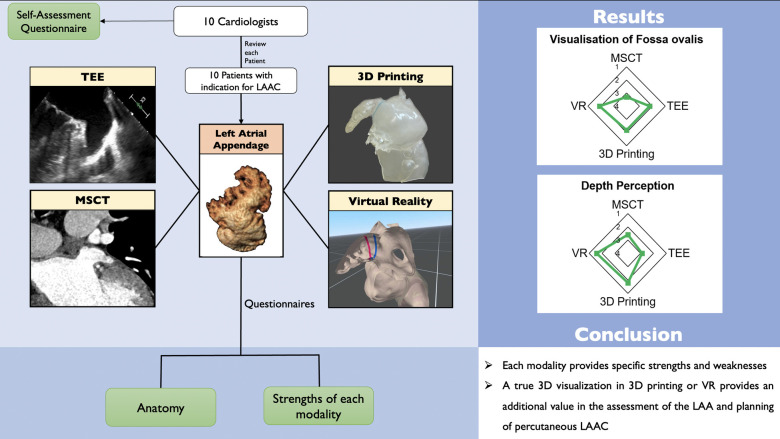

Comparison of conventional and advanced imaging techniques for left atrial appendage closure. Application of different imaging techniques for the visualization of the LAA: Four different modalities (TEE, MSCT, 3D printing, and VR) of 10 patients with indication for LAAC were each reviewed by 10 cardiologists or cardiologists in training. Questionnaires about the patient specific anatomy and the performance of the modality were answered. LAAC—left atrial appendage closure; MSCT—multi-slice computed tomography; TEE—transesophageal echocardiography; VR—virtual reality; LZ—landing zone.

## Introduction

Advances in cardiovascular imaging are a cornerstone in the development of transcatheter structural heart interventions (1). Among these, left atrial appendage (LAA) closure (LAAC) has been established as a safe and effective procedure for stroke prevention in patients with atrial fibrillation unsuitable for oral anticoagulation (2, 3). Imaging plays a key role in a comprehensive understanding of the highly variable, three-dimensional (3D) anatomy of the LAA (4). There is an ongoing debate on the value of different imaging modalities: traditionally, transesophageal echocardiography (TEE) has been the preprocedural and intraprocedural modality of choice (5). The major strength of TEE is intraprocedural real time guidance of key procedural steps with high temporal resolution (5). However, contrast-enhanced multi-slice computed tomography (MSCT) is increasingly utilized for preprocedural planning, enabling accurate device sizing due to high spatial resolution (6). Despite the use of these imaging techniques, fully grasping the intricate LAA anatomy may be challenging (5). However, a precise understanding of the patient-specific anatomy is crucial in order to prevent complications and to minimize the number of procedures with failed implantation success (7). Furthermore, adequate preprocedural imaging may reduce deployment attempts and the average number of devices used per patient (8). Advanced technologies offering true 3D visualization of the LAA have gained attention recently (9–11). Among these, 3D printing may precisely visualize patient-specific anatomy of the left atrium and LAA (12) and has shown to improve procedural parameters in LAAC (13). Virtual reality (VR) allows immersion and represents an alternative method of true 3D visualization (14–17) and additionally allows direct interaction (such as measurements) and stereoscopic depth perception of the virtual model (18, 19).

Comparative strengths and weaknesses of these imaging techniques for LAAC remain unknown. This study aimed to assess the value of conventional (TEE, MSCT) and advanced imaging modalities (3D printing, VR) for cardiologists in preparation for percutaneous LAAC.

## Methods

### Study design and questionnaires

In this retrospective single-center study, ten patients with non-valvular atrial fibrillation and indication for LAAC were included. The study was approved by the local ethics committee and complies with the 1975 Declaration of Helsinki.

All patients underwent screening and percutaneous LAAC with an Amplatzer Amulet occluder device at the University Hospital of Duesseldorf, Germany. Routine imaging included preprocedural MSCT, intraprocedural TEE and fluoroscopy: MSCT was used for preprocedural planning of the procedure, device sizing, and exclusion of LAA thrombi, procedural guidance was performed by TEE and fluoroscopy. 3D printings and VR models of all patients were fabricated based on MSCT data. Thus, the cardiac anatomy of all ten patients was available in four imaging modalities.

All MSCT, TEE, 3D printing, and VR datasets were reviewed by ten board-certified cardiologists/cardiologists in training with extensive experience in cardiovascular imaging. All participants answered a self-assessment questionnaire, then reviewed MSCT and TEE on two-dimensional (2D) screens, manually/visually assessed 3D printings and VR models. They answered a questionnaire about the patient-specific anatomy directly after each modality; after reviewing all studies and modalities, the participants answered a general questionnaire regarding the comparison of modalities. Details on the questionnaires are reported below.

### Multi-slice computed tomography: image acquisition and analysis

All patients received a pre-procedural contrast-enhanced, ECG-gated MSCT (Siemens Healthineers, Erlangen, Germany, “SOMATOM Definition Edge”). The temporal resolution was 150 ms. The collimation of the detector was 128 × 0.6 mm. Contrast agent (70–90 ml) was injected in a peripheral vein. The scan was performed with a delay of approximately 1 min after contrast injection to receive an optimal contrast enhancement of the LAA. All MSCT datasets were saved as Digital Imaging and Communications in Medicine (DICOM) files, transferred to a workstation, and analysis was performed using dedicated software (3mensio Structural Heart™, Pie Medical Imaging BV, Maastricht, The Netherlands).

Each cardiologist independently analyzed MSCT images. First, the LAA was semiautomatically located and displayed in multiplanar reconstruction (MPR). Then, the circumflex artery and coumadin ridge were marked manually to obtain the ostium plane. The landing zone (LZ) was defined as the plane 10–12 mm distal from the ostium, orthogonal to the long axis of the LAA. LAA depth was defined as a perpendicular line from the ostium to the LAA roof. The LAA and surrounding structures were evaluated using MPR, 3D reconstruction, and by scrolling through two-dimensional axial planes.

### Transesophageal echocardiography

All TEE studies were performed under conscious sedation using an EPIQ 7C (Philips, Amsterdam, Netherlands) ultrasound machine. TEE was performed by cardiologists with extensive experience in periprocedural guidance of structural heart interventions. The LAA was visualized in 0°, 45°, 90° and 135° from a midesophageal view. The ostium was defined using MSCT as the line between the tip of the coumadine ridge and the circumflex artery. The LZ was defined as the line 10 mm distal from the ostium perpendicular to the long axis of the LAA. Final device sizing was based on multimodality imaging including MSCT and TEE.

### Three-dimensional printing

A three-dimensional model was generated based on high-quality contrast-enhanced MSCT datasets using the commercially available software platform Mimics (Materialise, Leuven, Belgium). All patients had sufficient MSCT image quality. DICOM files were uploaded, a semi-automatic segmentation of blood vessels and left and right atrium with the interatrial septum was performed by Materialise. This was followed by a thorough manual segmentation by a company employee under the guidance of an imaging expert from our institution with extensive experience in the evaluation of MSCTs. Then, the dataset was exported in Standard Tessellation Language (STL) format. After final adjustments, materials and colors were selected. In our case, we selected the HeartPrint Flex model (Materialise, Leuven, Belgium) with flexible materials consisting of Agilus polymers and silicone offering a realistic representation of tissue properties. Subsequently, manufacturing of the printed model was initiated and the printed model was sent to our institution by mail. All MSCTs were successfully converted in high quality 3D printing models.

### Virtual reality

Visualization in VR was performed using the same software platform as for 3D printing (Mimics, Materialise, Leuven, Belgium): VR was based on high-quality MSCT images. DICOM datasets were uploaded and a 3D model was generated. The Meta Quest 2 (Meta, Irvine, California, USA) wireless VR headset was used for visualization in VR with a Single Fast-Switch LC display (1,832 × 1,920 pixels per eye) with a refresh rate of 72 Hz. Movement was enabled with six degrees of freedom (three rotational, three translational planes). Two handheld controllers allowed interaction with the 3D heart model, and specific tools allowed zoom, grab, rotate, and slice through the 3D model. Overviews of the virtual heart model and specifically the LAA and the surrounding structures were evaluated, then, by rotating the model and slicing through the atria, and zooming in, the examiner “walked into” the left atrium and appreciated a detailed view of the LAA and surrounding structures. Measurements done in the MSCT dataset (ostium, LZ) were displayed in the virtual model. Furthermore, a previously chosen optimal site for transseptal puncture was displayed in VR.

### Questionnaires

The questionnaires addressed the following topics: First, all participants answered a self-assessment questionnaire, which assessed the level of experience in general cardiology, interventional cardiology, and different cardiovascular imaging modalities (transthoracic and transesophageal echocardiography, MSCT, magnetic resonance imaging); level of experience with advanced imaging modalities such as VR and 3D printing were also queried. Second, participants rated visualization of the anatomy in each modality subjectively. Here, visualization of the coronary arteries (circumflex artery), ostium, and LZ measurements, device sizing, understanding of 3D anatomy and neighboring structures, and visualization of fossa ovalis was rated. Additionally, an objective assessment of the respective anatomy of each patient was performed. In brief, the LAA morphology, number of lobes, orientation of the LAA, shape of the ostium, septum thickness, dimensions of the left and right atrium, and the optimal transseptal puncture site were assessed. Correct answers were rated with one point, and incorrect answers with zero points. Third, following the review of all patients, each participant answered one questionnaire for each modality. Here, participants rated the efficiency and usefulness, the practicability, and the inherent uncertainty of each modality.

In order to achieve consistency among answers, mainly short and clearly formulated questions were used. Furthermore, similar structured and worded questions were applied. Objective anatomy questionnaires consisted of multiple-choice questions with one single best answer. All other questions were answered on a five-point Likert scale (with 1 = strongly agree, 2 = agree, 3 = neither disagree, nor agree, 4 = disagree, and 5 = strongly disagree).

### Statistics

Categorical variables are displayed as percentages. Likert scale responses are reported as mean ± standard deviation and parametric statistics were applied as described by Dr. Geoff Norman (20). Thus, multiple comparisons were analyzed using ANOVA with Bonferroni correction. *P*-values < 0.05 were considered statistically significant. GraphPad Prism version 9 (GraphPad Software, San Diego, USA) was used for all calculations.

## Results

### Participants

**Figure 1:**
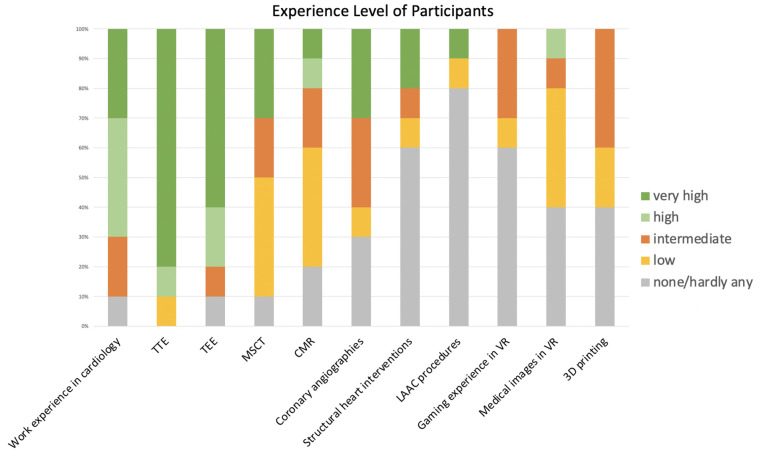
Clinical experience of participants. Overview of the clinical experience of participating cardiologists. Participants had varying degrees of experience in cardiology in general, but also in interventional cardiology and cardiovascular imaging. Most participants had little or no experience in VR or 3D printing. VR, virtual reality; TTE, transthoracic echocardiography; TEE, transesophageal echocardiography; MSCT, multi-slice computed tomography; CMR, cardiovascular magnetic resonance; LAAC, left atrial appendage closure.

Data from 10 patients were evaluated by our structural heart disease team. The team comprised of experienced interventionalists and interventional imagers with advanced imaging experience as well as two fellows in training with advanced imaging experience. 70% of the participants were male; 70% had >6 years of work experience in cardiology. Most participants had extensive experience in echocardiography; three were very experienced in MSCT (>1,000 exams), while half of the participants had evaluated <100 MSCTs. There was not much experience in the evaluation of either medical images in VR or of 3D-printed hearts. Due to the investigation in a real-world setting, there is a certain heterogeneity in the experience with the individual modalities among the participants of the present study. Further details are displayed in Figure 1.

### Spatial orientation, manual handling and inherent uncertainty

Among all modalities, VR and 3D printing offered the best depth perception, as the main component of 3D vision and distance estimation. The manual handling was rated highest in TEE and VR (TEE: 1.67 ± 0.5; VR: 2.0 ± 1.0) compared to MSCT and 3D printing (MSCT: 2.67 ± 1.4; 3D printing: 2.56 ± 1.1). In terms of simplicity of use, no significant difference was observed among the modalities. The learning curve and the trust in the modality were also comparable. However, when asked which modality the participants would trust as a sole modality for LAAC planning, MSCT and TEE were rated high, while participants would not rely on 3D printing or VR.

### Subjective assessment of anatomy

The ostium measurements were rated most reliable in MSCT compared to other modalities. Similar findings were observed for LZ measurements. Overall, participants trusted device sizing in MSCT the most. However, among the modalities, MSCT was least accurate in the visualization of the fossa ovalis, while the other modalities performed similarly. Furthermore, MSCT, 3D printing, and VR offered the most comprehensive understanding of the spatial relation to neighboring anatomic structures, while TEE was not useful in this regard. The modalities showed no significant difference in the visualization of intracardiac structures. Extracardiac structures were least accurately visualized in TEE, while the other modalities did not show a significant difference. The course of the coronary arteries was best visualized in MSCT and worst in TEE. Further details are displayed in Table 1.

**Table 1:** Subjective assessment of the anatomy.

	MSCT	TEE	MSCT vs. TEE	3DP	MSCT vs. 3DP	VR	MSCT vs. VR
					*p*-value		*p*-value
			*p*-value				
Visualization of coronary arteries	1.9 ± 1.0	3.9 ± 1.1	*p* < 0.01	2.6 ± 1.6	*p* < 0.01	2.7 ± 1.6	*p* < 0.01
Reliability of ostium measurements	1.6 ± 0.7	2.4 ± 1.1	*p* < 0.01	2.2 ± 1.0	*p* < 0.01	2.1 ± 1.1	*p* = 0.02
Reliability of LZ measurements	1.8 ± 0.8	2.5 ± 1.1	*p* < 0.01	2.4 ± 1.1	*p* < 0.01	2.3 ± 1.1	*p* < 0.01
Device sizing	1.9 ± 0.8	2.6 ± 0.9	*p* < 0.01	2.5 ± 1.0	*p* < 0.01	2.5 ± 1.1	*p* < 0.01
3D understanding of neighboring structures	1.9 ± 0.9	2.9 ± 1.2	*p* < 0.01	2.1 ± 1.1	*p* = 0.96	1.9 ± 1.1	*p* > 0.99
Visualization of intracardiac structures	1.9 ± 0.7	2.2 ± 1.1	*p* > 0.99	1.7 ± 0.5	*p* > 0.99	1.6 ± 0.5	*p* > 0.99
Visualization of extracardiac structures	1.7 ± 0.7	3.4 ± 1.1	*p* = 0.015	2.9 ± 1.1	*p* = 0.18	3.1 ± 1.4	*p* = 0.07
Visualization of fossa ovalis	3.3 ± 1.4	2.2 ± 1.3	*p* < 0.01	2.2 ± 1.4	*p* < 0.01	1.9 ± 1.3	*p* < 0.01

Results from the Likert scale questionnaire presented as mean ± SD from 1 point (strongly agree) to 5 points (strongly disagree). *P*-values for the correlation of answers with current gold standard MSCT. MSCT, multi-slice computed tomography; TEE, transesophageal echocardiography; 3DP, three-dimensional printing; VR, virtual reality.

### Objective assessment of anatomy

In the multiple-choice questionnaire evaluation of the patient-specific anatomy, the accuracy to identify the correct LAA morphology (cactus, chicken wing, wind sock, cauli flower) was similar among the modalities. However, the number of lobes was poorly identified with TEE, while all other modalities performed similarly. Likewise, the orientation of the LAA and the shape of the ostium were least accurately identified in TEE. The left and right atrial dimensions were more precisely identified in TEE and MSCT, compared to 3D printing and VR. However, this difference was not statistically significant. Concerning the characterization of the interatrial septum (IAS) morphology, TEE outperformed the other modalities. TEE most accurately identified the IAS anatomy and thickness. However, no difference was observed in identifying the optimal transseptal puncture site among the modalities. Further details are displayed in Table 2.

**Table 2:** Objective assessment of the anatomy.

	MSCT	TEE	MSCT vs. TEE	3DP	MSCT vs. 3DP	VR	MSCT vs. VR
			*p*-value		*p*-value		*p*-value
LAA morphology (cactus, chicken wing, wind sock, cauli flower)	45%	35.6%	*p* = 0.99	40%	*p* > 0.99	37%	*p* > 0.99
Number of lobes	59%	32.2%	*p* = 0.0013	51%	*p* > 0.99	54%	*p* > 0.99
Orientation of the LAA	53%	32.2%	*p* = 0.023	64%	*p* = 0.56	69%	*p* = 0.1
Shape of the ostium	76%	55.6%	*p* = 0.011	71%	*p* > 0.99	78%	*p* > 0.99
LA dimensions	56%	47.8%	*p* > 0.99	38%	*p* = 0.1	37%	*p* = 0.052
RA dimensions	53%	56.7%	*p* > 0.99	36%	*p* = 0.15	38%	*p* = 0.23
IAS thickness	55%	77.8%	*p* = 0.007	56%	*p* > 0.99	60%	*p* > 0.99
Transseptal puncture site	47%	46.7%	*p* > 0.99	48.9%	*p* > 0.99	48%	*p* > 0.99

Results from the questionnaire are presented as percentages of correct answers. *P*-values for the correlation of answers with current gold standard MSCT. MSCT, multi-slice computed tomography; TEE, transesophageal echocardiography; 3DP, three-dimensional printing; VR, virtual reality; RA, right atrium; LA, left atrium.

### Hypothetical impact on LAAC

**Figure 2:**
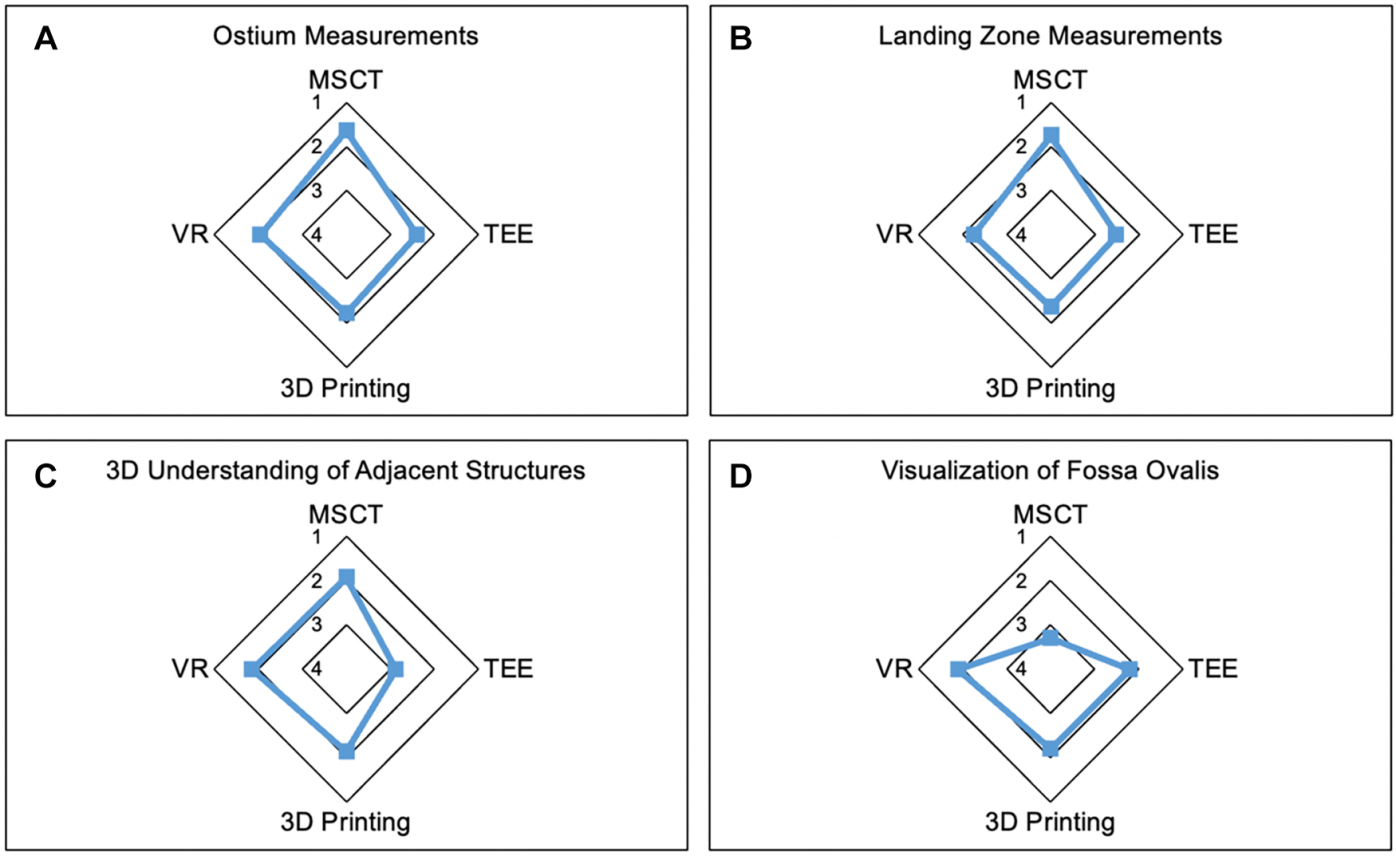
Strengths of the modalities. Radar Chart display of results from the Likert scale questionnaire presented as mean values from 1 point (strongly agree) to 5 points (strongly disagree). (**A,B**) demonstrate the most reliable measurements of ostium (**A**) and LZ (**B**) dimensions in MSCT. (**C**) shows the lowest accuracy in visualization of adjacent anatomic structures in TEE compared to the other modalities. (**D**) Least accuracy for visualization of the fossa ovalis was observed in MSCT. MSCT, multi-slice computed tomography; VR, virtual reality; TEE, transesophageal echocardiography.

**Figure 3:**
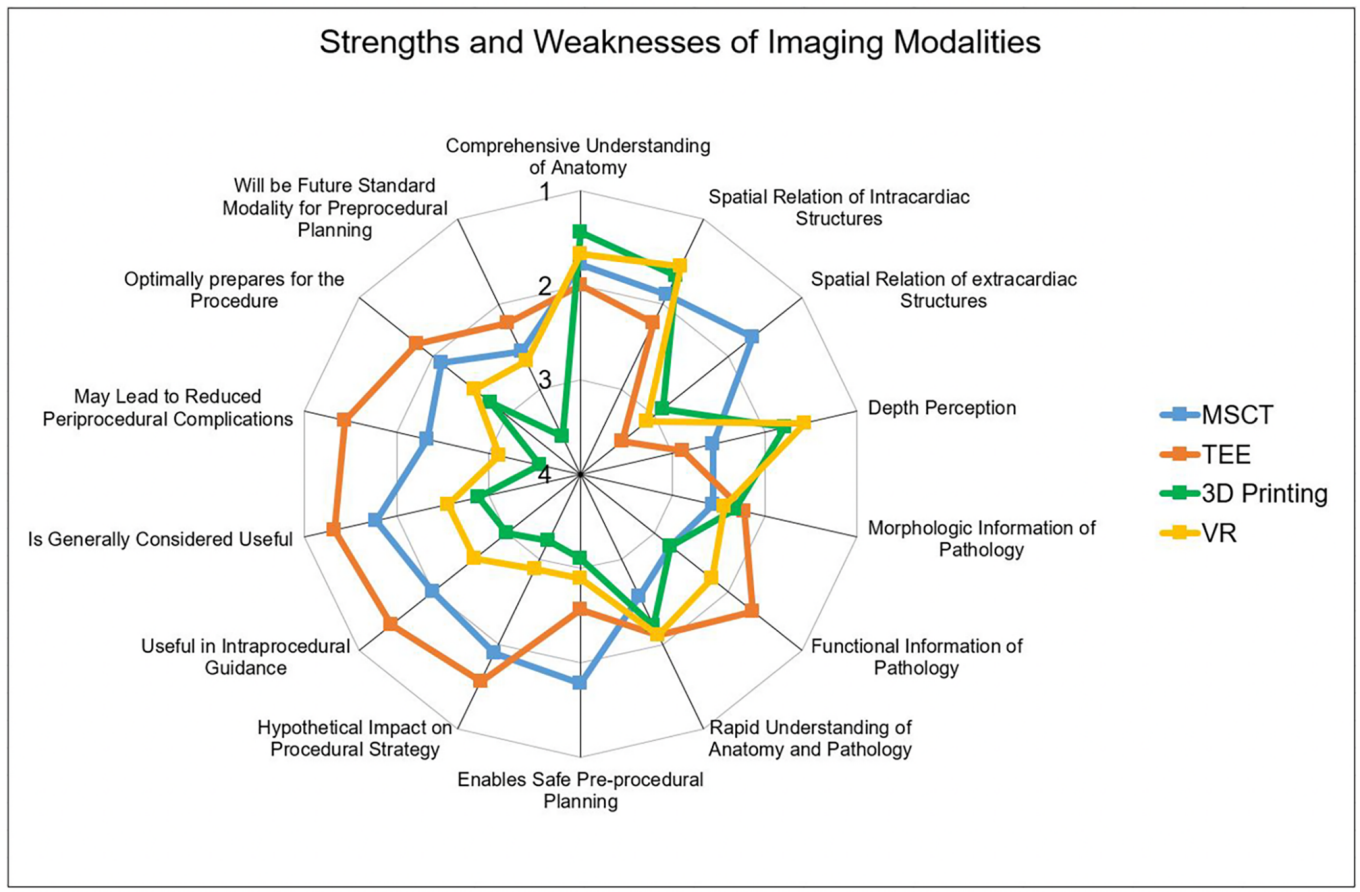
Performances of the imaging modality. Radar Chart display of results from the Likert scale questionnaire presented as mean values from 1 point (strongly agree) to 5 points (strongly disagree). While MSCT (**A**) was rated best for evaluation of extracardiac structures, TEE (**B**) was the strongest modality offering functional information and was rated most useful for procedural guidance. 3D printing (**C**) and VR (**D**) outperformed the conventional modalities concerning depth perception. MSCT, multi-slice computed tomography; VR, virtual reality; TEE, transesophageal echocardiography.

As this was a retrospective study, the influence of the different modalities on procedural steps were not directly investigated. However, to address this point, several questions regarding the hypothetical impact on the procedure were implemented. It could be shown, that all modalities offer comparable morphologic information. However, TEE best displayed functional information such as myocardial or valvular function compared to other modalities. Furthermore, participants rated MSCT the highest in the capability to enable safe procedure planning. Additionally, TEE was rated most useful for intraprocedural guidance, while the benefit of VR and 3D printing in this regard was rated low. Finally, participants rated the hypothetical impact on the procedural strategy. Here, MSCT and VR were rated higher compared to VR and 3D printing. Detailed performances of the modalities are displayed in Figures 2, 3.

## Discussion

This study demonstrates a side-by-side comparison of the current imaging gold standard for LAAC (MSCT and TEE) with advanced techniques such as 3D printing and VR visualization for LAAC. To our knowledge, this is the first study to directly compare these four modalities in the assessment of the LAA. The main findings are as follows: in the eyes of participants experienced in cardiovascular imaging, each modality provides specific strengths and weaknesses. MSCT with its high spatial resolution provides detailed information about the patient specific anatomy and represents the strongest modality for device sizing. TEE provides functional information and offers optimal guidance of key procedural steps. 3D printing and VR offered the best three-dimensional understanding of the anatomy due to the superior depth perception. Thus, a true 3D visualization provides an additional value in the evaluation of the LAA.

MSCT and TEE are the two conventional imaging modalities recommended for preprocedural imaging for LAAC (21). Both provide a comparable accuracy for identification of LAA thrombi (22). However, MSCT is superior to TEE regarding spatial resolution. Thus, MSCT enables more precise measurements of the LAA using multiplanar reconstruction, while 2D TEE tends to underestimate ostium and LZ dimensions (23). This is in line with the findings of the present work. Participants of our study acknowledge the capability of MSCT to precisely display the endocardial border of the LAA enabling accurate device sizing. Moreover, MSCT was best rated for visualization of extracardiac surrounding structures, as well as structures attached to the LAA such as the coronary arteries. Here, especially the circumflex artery (CX) is of importance due to its proximity to the LAA. CX obstruction following percutaneous LAAC has been reported (24), although very rare. Precise knowledge of the course of the coronary artery may prevent this complication.

The major strength of TEE is the ability to guide procedural steps during LAAC (25). A key step during the procedure is the transseptal puncture (TSP). TSP aims to achieve a coaxial alignment with the long axis of the LAA. This is usually achieved with an inferior-posterior puncture site and can be guided accurately with TEE (4). However, individual anatomy may vary and demand a different TSP site. Moreover, TSP is associated with potentially fatal complications such as cardiac tamponade or puncture of the aortic root (26). Guidance with TEE has shown to reduce these complications (27). Accordingly, participants of the present study rated TEE, together with VR, the highest in the capability to display the IAS with the fossa ovalis. Moreover, participants correctly characterized the IAS most frequently in TEE. This is of importance since IAS characteristics such as thickness, fibrosis, and mobility can predict the complexity of TSP (28). Furthermore, participants rated TEE as the modality most likely to be associated with a reduced complication rate. However, TEE has major limitations, especially concerning LAA sizing. Particularly 2D TEE underestimates ostium and LZ dimensions (29). *So* et al. demonstrated, that preprocedural MSCT in addition to TEE is associated with a higher success rate of LAAC, shorter procedural time, and lower number of changes in device size during the procedure (30). Furthermore, the capability of TEE to visualize neighboring structures is limited. Spatial orientation requires a great deal of experience and cognitive processes, especially in 2D. Detailed questions about the 3D anatomy, such as the number of lobes of the LAA, are difficult to evaluate in 2D TEE. Finally, these conventional imaging techniques either acquire 2D images or display 3D datasets on flat screens.

Various 3D visualization techniques have been studied to provide comprehensive 3D visualization of complex patient-specific anatomy. Among these, 3D printing is the most widely studied technology for LAAC. Its use in the setting of LAAC was associated with improved device sizing, less peri-device leakage, and reduced procedure time (13, 31). Furthermore, 3D printing was shown to offer a superior understanding of spatial relations of intra- and extracardiac structures (32). In line with these findings, participants of the present study perceived 3D printing as specifically valuable for the visualization of the relation of intracardiac structures and better understanding of patient anatomy. Furthermore, participants most often identified LAA orientation correctly in 3D printing and VR. However, the 3D printing replica applied in this study offered limited information about extracardiac structures by prior segmentation. Hence, extracardiac structures could insufficiently be assessed. Furthermore, due to the isolation of the region of interest, participants were highly erroneous in judging left and right atrial size, since no reference structures (such as the ventricles) were depicted. However, the major benefit of 3D printing is the free movement of the model and the haptic feedback, depending on the material applied (33). Nonetheless, major limitations of 3D printing remain high fabrication costs, the duration of production as well as the time-consuming segmentation needed. VR may overcome some of these limitations since it allows full immersion into the 3D virtual environment (34, 35). Free movement, zooming, and cropping of the VR model allow precise appreciation of the patient-specific anatomy. As in 3D printing, participants of the present study acknowledged the superior depth perception and thus a true 3D visualization. Overall, 3D printing and VR showed similar strengths and weaknesses in the present study. This is consistent with the results of a study by Lau et al. where the authors found no significant difference between 3D printing and VR in terms of diagnostic skills, education or preoperative planning in a cohort of congenital heart disease patients (36). To some extent, this might be explained by the similar preprocessing steps of the 3D printed replica and VR application utilized here. However, Interestingly, despite the multitude of advantages offered by a true 3D visualization, participants rated MSCT and TEE higher in the ability to prepare for a procedure. This may be explained to some extent by inherent uncertainty and skepticism towards advanced technologies due to lack of experience. To overcome this, it can be an important factor in the future to implement these technologies in everyday clinical practice.

### Study limitations

The present study has some limitations. First, participants were cardiologists and cardiologists in training with extensive experience in cardiovascular imaging. However, only one participant had a high level of experience in the performance of LAAC as an interventionalist. Another participant performed less than ten LAAC procedures. Thus, the interventionalists view remains insufficiently represented in the present study. A further study including this group of cardiologist may provide additional information. Second, only a small number of patients and only patients with suitable anatomy and sufficient image quality with successful LAAC were included in the study. The aim was to prevent an influence of the image quality on the overall assessment of the individual modalities. Last, the application of a Likert scale may be subject to response bias and central tendency bias which may affect validity of the data.

## Conclusion

This side-by-side comparison highlights distinct strengths and weaknesses of different imaging modalities in the setting of left atrial appendage closure. In addition to conventional imaging methods, advanced modalities add information and can potentially improve procedure planning. Clinical studies are needed to evaluate whether a more comprehensive understanding through advanced multimodal imaging of patient-specific anatomy may be translated into improved procedural outcomes.

## Data Availability

The original contributions presented in the study are included in the article/Supplementary Material, further inquiries can be directed to the corresponding author.
